# Cytosolic 10-formyltetrahydrofolate dehydrogenase regulates glycine metabolism in mouse liver

**DOI:** 10.1038/s41598-019-51397-1

**Published:** 2019-10-17

**Authors:** Natalia I. Krupenko, Jaspreet Sharma, Peter Pediaditakis, Baharan Fekry, Kristi L. Helke, Xiuxia Du, Susan Sumner, Sergey A. Krupenko

**Affiliations:** 1 Nutrition Research Institute, University of North Carolina, Chapel Hill, NC USA; 2 Department of Nutrition, University of North Carolina, Chapel Hill, NC USA; 3 Department of Comparative Medicine, Medical University of South Carolina, Charleston, SC USA; 4 Department of Bioinformatics & Genomics, UNC Charlotte, Charlotte, NC USA; 5 Present Address: Institute of Molecular Medicine, McGovern Medical School, University of Texas Health Science Center, Houston, TX USA

**Keywords:** Enzymes, Metabolomics, Biochemistry, Tumour-suppressor proteins

## Abstract

ALDH1L1 (10-formyltetrahydrofolate dehydrogenase), an enzyme of folate metabolism highly expressed in liver, metabolizes 10-formyltetrahydrofolate to produce tetrahydrofolate (THF). This reaction might have a regulatory function towards reduced folate pools, *de novo* purine biosynthesis, and the flux of folate-bound methyl groups. To understand the role of the enzyme in cellular metabolism, *Aldh1l1*^−/−^ mice were generated using an ES cell clone (C57BL/6N background) from KOMP repository. Though *Aldh1l1*^−/−^ mice were viable and did not have an apparent phenotype, metabolomic analysis indicated that they had metabolic signs of folate deficiency. Specifically, the intermediate of the histidine degradation pathway and a marker of folate deficiency, formiminoglutamate, was increased more than 15-fold in livers of *Aldh1l1*^−/−^ mice. At the same time, blood folate levels were not changed and the total folate pool in the liver was decreased by only 20%. A two-fold decrease in glycine and a strong drop in glycine conjugates, a likely result of glycine shortage, were also observed in *Aldh1l1*^−/−^ mice. Our study indicates that in the absence of ALDH1L1 enzyme, 10-formyl-THF cannot be efficiently metabolized in the liver. This leads to the decrease in THF causing reduced generation of glycine from serine and impaired histidine degradation, two pathways strictly dependent on THF.

## Introduction

Folate coenzymes participate in numerous biochemical reactions of one-carbon transfer^1^. The network of folate utilizing reactions is referred to as one-carbon metabolism with more than two dozen enzymes participating in folate conversions. These reactions are involved in metabolic pathways of amino acid biosynthesis and degradation, *de novo* nucleotide biosynthesis, formate clearance, and the regulation of protein biosynthesis in mitochondria^1^. The role of folate pathways in energy balance in the cell was also underscored^2^. Higher animals including humans cannot synthesize folate and instead must obtain it from their diet. Folate is indispensable for normal cell function and insufficient dietary folate intake, as well as deregulation of folate metabolism, is associated with several diseases most notably neural tube defects^3^. Studies of mice with knockouts of folate-related genes have shown that the loss of certain key folate-metabolizing enzymes is embryonically lethal, the effect associated with the crucial role of folate pathways for nucleic acid biosynthesis and methylation processes^4–10^.

One of the folate-metabolizing enzymes, ALDH1L1 (10-formyltetrahydrofolate dehydrogenase) is a major cytosolic protein in the liver but its precise biological significance is not clear^11^. The enzyme converts 10-formyl-THF to THF and CO_2_ in an NADP^+^-dependent reaction. This reaction could be important for replenishing the cellular THF pool, which is involved in several metabolic processes in the cell including serine to glycine conversion, histidine degradation, and formate oxidation^11,12^. ALDH1L1 could also regulate purine levels by competing for 10-formyl-THF, which is a substrate for two reactions in the *de novo* purine pathway. Furthermore, the enzyme clears one-carbon groups, in the form of CO_2_, from the folate pool which might limit the overall biosynthetic capacity of folate-dependent reactions thus playing a regulatory role^11,13^. In support of such proliferation regulatory role, we have recently shown that ALDH1L1 is down-regulated in S-phase of the cell cycle in NIH 3T3 cells^14^. Several studies also implicated ALDH1L1 as a folate depot, the function likely important for preventing folate degradation^15–17^. Finally, the enzyme can be important as a source of NADPH, the role highlighted for the mitochondrial 10-formyltetrahydrofolate dehydrogenase isozyme^2^.

Of note, ALDH1L1 belongs to the family of aldehyde dehydrogenases (ALDH)^18^. The C-terminal domain of the protein has sequence homology with several members of the ALDH family, has a typical ALDH fold and catalyzes *in vitro* aldehyde dehydrogenase reaction using short-chain aldehydes as substrate^11^. Though natural aldehyde substrates of ALDH1L1 are not known, such function of the enzyme cannot be excluded at present.

Thus, while the biochemical reactions catalyzed by ALDH1L1 are well characterized, the effect of the enzyme on overall cellular metabolism was not addressed. In support of this notion, a recent study has also implicated ALDH1L1 in the conversion of dihydrofolate to folic acid through an unclear mechanism^17^. Importantly, the enzyme is strongly down-regulated in cancer cell lines and malignant tumors^19,20^ through the promoter methylation^21^ but its role in tumorigenesis and tumor development is not fully understood (reviewed in^12,22^). In the present study, we have generated *Aldh1l1* knockout mice and characterized their reproductive ability, phenotype and the effect of the gene loss on the liver metabolic profile, reduced folate pools and expression of inflammation-related genes. Our study provides experimental evidence that ALDH1L1 regulates reduced folate pools as well as glycine metabolism in the liver.

## Results

### Generation and characterization of *Aldh1l1*^−/−^ mice

**Figure 1:**
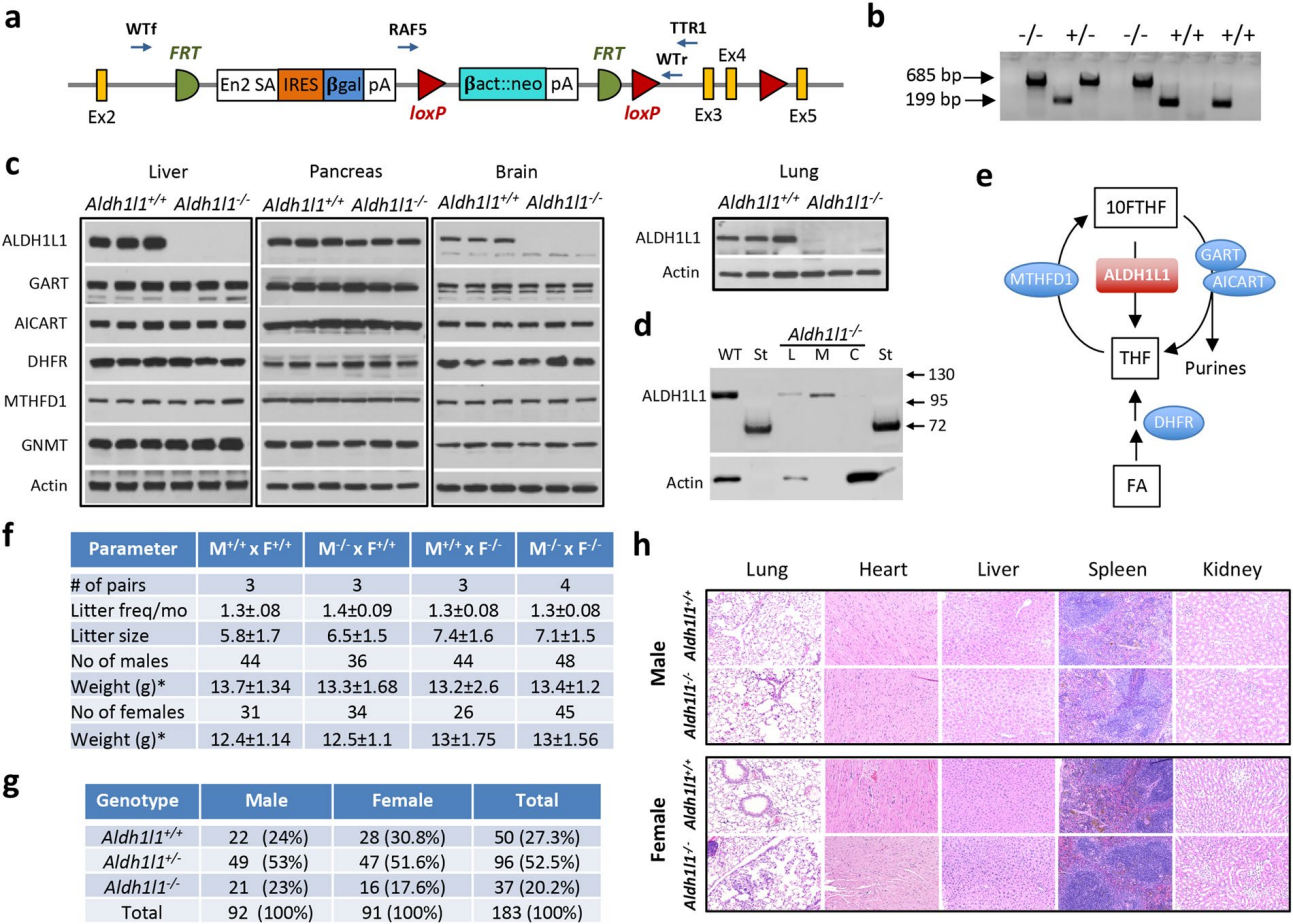
Characterization of *Aldh1l1* knockout mice. (**a**) Schematic presentation of the *Aldh1l1* gene-trapping vector. *Ex*, exon; *FRT*, Flp-recombinase target; *loxP*, Cre-recombinase site. Primers for mouse genotyping and their approximate target locations are shown (WTf and WTr, primers for the wild type allele; RAF5 and TTR1, primers for the disrupted allele). (**b**) Genotyping pattern for *Aldh1l1*^−/−^, *Aldh1l1*^+/−^ and *Aldh1l1*^+/+^ mice (two PCR reactions were run for each DNA sample, for the detection of the wild type allele with the WTf/WTr primer pair and for disrupted allele with the RAF5/TTR1 primer pair; corresponding PCR products are 199 bp and 685 bp). (**c**) Western blot assays of *Aldh1l1*^+/+^ and *Aldh1l1*^−/−^ mouse tissues (original full-size blot images are shown in Supplementary Fig. S1). (**d**) Western blot assay of lysate from *Aldh1l1*^+/+^ (WT) and *Aldh1l1*^−/−^ (L) pancreas; mitochondrial (M) and cytosolic (C) fractions isolated from KO mouse; St, standards. (**e**) Schematic depicting the conversion of 10-formyl-THF (10FTHF) and THF by corresponding enzymes (MTHFD1, methylenetetrahydrofolate dehydrogenase 1; GART, phosphoribosylglycinamide formyltransferase; AICART, phosphoribosylaminoimidazolecarboxamide formyltransferase; DHFR, dihydrofolate reductase). (**f**) Fecundity, litter size weight and male/female numbers for different combinations of breeder genotypes. (**g**) Sex and genotype distribution of progeny from intercrosses of *Aldh1l1*^+/−^ mice. For panels f and g data are expressed as mean ± SD. (**h**) H&E staining of tissue sections from *Aldh1l1*^+/+^ and *Aldh1l1*^−/−^ mice.

We have generated *Aldh1l1*^−/−^ mice using ES cells obtained from the KOMP consortium. These cells have a “knockout first” *Aldh1l1* gene alteration generated via homologous recombination with the gene-trapping vector depicted in Fig. 1a. The trapping cassette was inserted in the intron upstream of exon 3 creating a constitutive null mutation. PCR-based genotyping of the wild type *Aldh1l1* allele generated a 199 bp fragment, whereas amplification of the disrupted allele generated a 685 bp fragment (Fig. 1b). The successful knockout of the *Aldh1l1* gene was demonstrated by the loss of the ALDH1L1 protein assessed by Western blot assays (Fig. 1c; full-size images are shown in Supplementary Fig. S1). Of note, heterozygous *Aldh1l1*^+/−^ mice have shown partial decrease in the Aldh1l1 protein level in the liver, suggesting a gene-dosage effect of the protein expression (Supplementary Fig. S2). Surprisingly, the band corresponding to ALDH1L1 was still seen in the pancreas of *Aldh1l1*^−/−^ mice though it had lower intensity (Fig. 1c). We tested whether this was caused by the cross-reactivity of our polyclonal antibody with mitochondrial isozyme ALDH1L2^23^, which sequence is 75.5% identical to the sequence of ALDH1L1. Of note, pancreas has the highest level of ALDH1L2 among all organs^23^. This was found to be the case: Western blot assays of cytosolic and mitochondrial fractions of pancreatic tissues have shown the absence of ALDH1L1 in the cytosol (Fig. 1d). We have also evaluated levels of a panel of enzymes relevant to metabolism of 10-formyl-THF (Fig. 1c,e; GNMT was included in the panel because it is one of the most abundant folate-relevant protein in the liver and it regulates the flux of one-carbon groups from folate pools towards methylation or nucleotide biosynthesis^24^; full-size images are shown in Supplementary Fig. S1). We did not observe a major effect of the *Aldh1l1* KO on the levels of tested proteins in three examined organs, liver, pancreas and brain (Fig. 1c).

Both female and male *Aldh1l1*^−/−^ mice were viable, fertile and developed normally with the total body weight similar to the wild type littermates at weaning (Fig. 1f) and at the age of 6 mo (Supplementary Fig. S3). We observed similar numbers of offspring from mating male or female *Aldh1l1*^−/−^ with *Aldh1l1*^+/+^ mice, as well as from mating mice with both sexes having either *Aldh1l1*^−/−^ or *Aldh1l1*^+/+^ genotype (Fig. 1f). Analysis of litter size from the intercross of *Aldh1l1*^+/−^ mice revealed that there was no significant variation from the Mendelian distribution ratio for male pups but was some deviation for female pups (Fig. 1g). Breeding experiments with a combination of genotypes (Fig. 1f), however, suggests that such deviation could be the result of an insufficient number of analyzed litters. We identified no phenotypic differences between *Aldh1l1*^−/−^ and *Aldh1l1*^+/+^ mice in terms of growth, body weight and food consumption, and knockout mice appeared healthy. Gross examination of the brain, lungs, kidney, liver, pancreas, heart and spleen did not reveal any noticeable differences in the organ size or morphology between genotypes (Supplementary Fig. S3). H&E staining and histological analysis of the tissues has further indicated the lack of differences between the two genotypes (Fig. 1h). Overall, our study demonstrated that phenotypically, the *Aldh1l1*^−/−^ genotype is undistinguishable from the *Aldh1l1*^+/+^ genotype.

### Levels of reduced folate in Aldh1l1 KO mice

**Figure 2:**
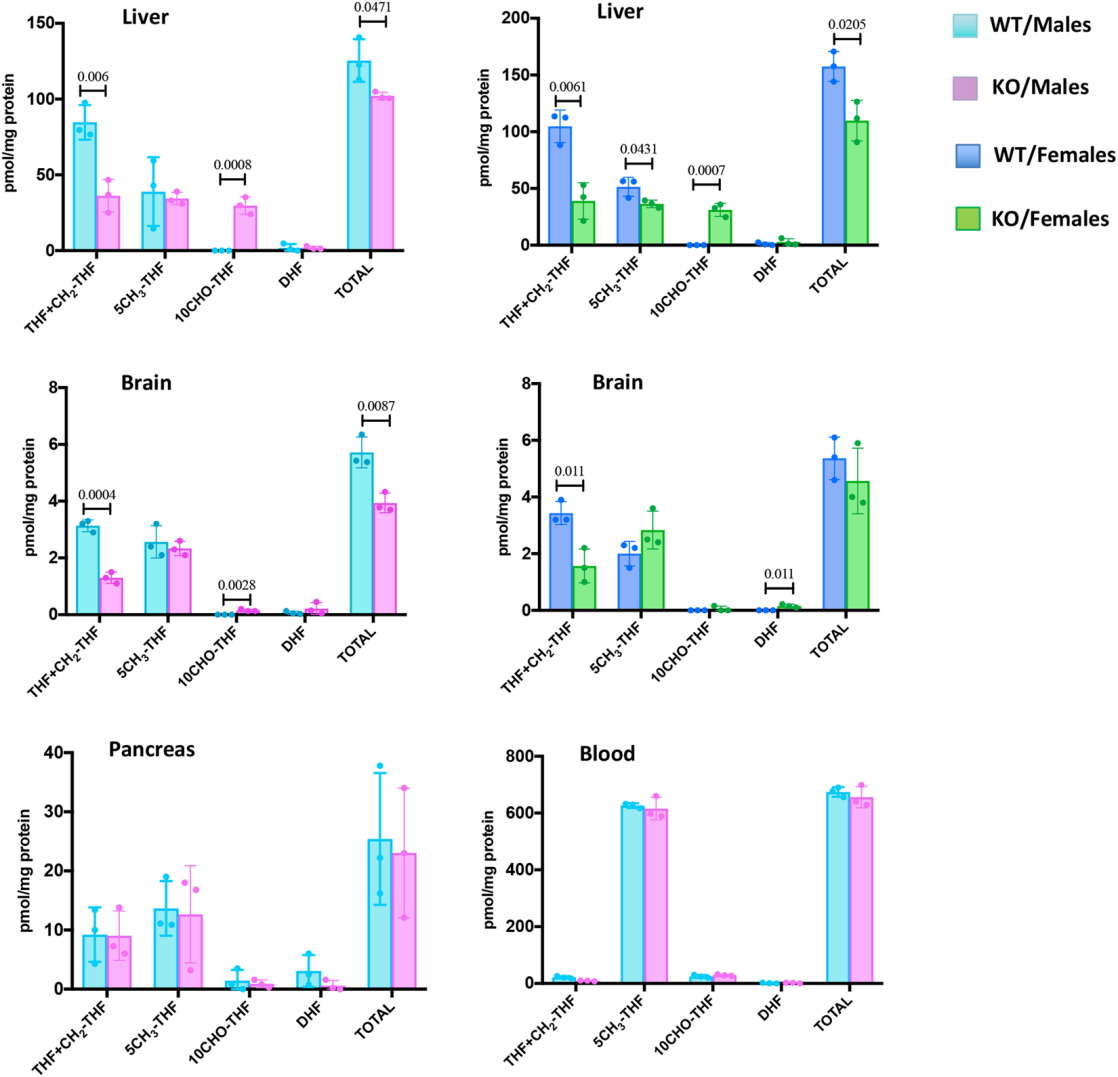
Levels of reduced folate pools in tissues of *Aldh1l1*^+/+^ and *Aldh1l1*^−/−^ male and female mice. For each group, samples from 3 mice were analyzed in quadruplicate. Statistical analysis was carried out using graph pad Prism VIII software. Data are expressed as mean ± SE. Statistical significance was calculated using Student’s t test.

We have further evaluated levels of reduced folate pools in livers of *Aldh1l1*^−/−^ and *Aldh1l1*^+/+^ mice. Our method allows the measurement of 10-formyl-THF, 5-methyl-THF, the combination of THF/5,10-methylene-THF, and the combination of dihydrofolate and folic acid^25,26^. In the liver of both male and female mice we have observed strong differences between the genotypes in two folate pools, 10-formyl-THF and THF/5,10-methylene-THF (Fig. 2). Other pools were not affected in the *Aldh1l1*^−/−^ genotype when compared to the *Aldh1l1*^+/+^ genotype (Fig. 2). These changes are in agreement with the role of ALDH1L1-catalyzed reaction (Fig. 1e). A statistically significant decrease in the total folate (1.2-fold for males and 1.4-fold for females) in the liver was also detected (Fig. 2), which could be associated with the proposed role for the ALDH1L1 protein as folate depot; in such a role, ALDH1L1 would protect reduced folate from degradation. Similar changes were observed in the brain of both sexes though the 10-formyl-THF pool in the brain was not increased as dramatically as in the liver (Fig. 2). Similar to the liver, the total folate was lower in the brain of knockout mice (though such difference for females was not statistically significant, Fig. 2). Of note, no changes in levels of folate in the pancreas or whole blood of male mice were observed between *Aldh1l1*^−/−^ and *Aldh1l1*^+/+^ genotypes (Fig. 2).

### *Aldh1l1*^*−/−*^ mice displayed metabolic symptoms of folate deficiency and have altered glycine metabolism

**Figure 3:**
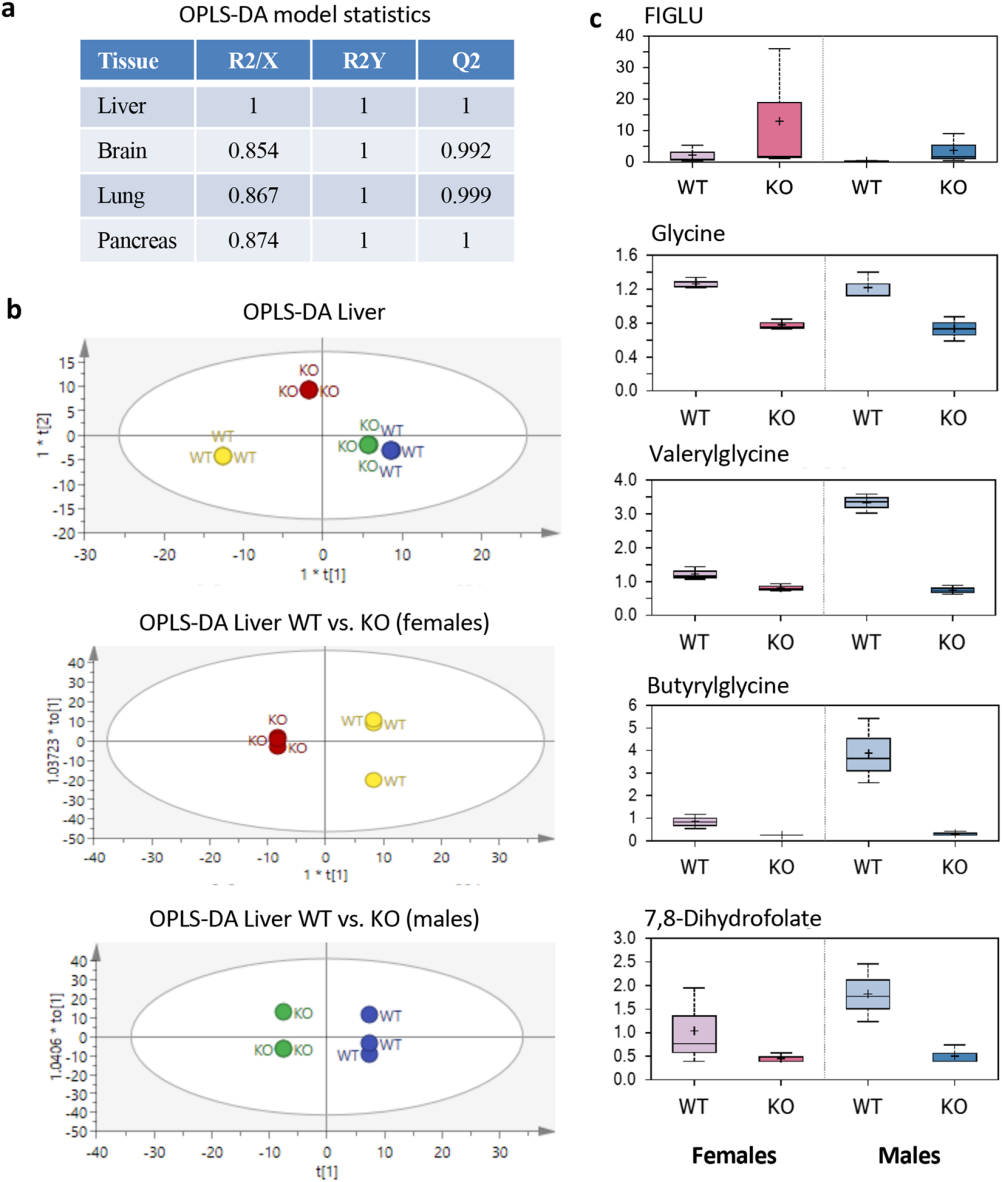
Analysis of metabolomic data of *Aldh1l1*^+/+^ and *Aldh1l1*^−/−^ male and female mouse liver. (**a**) OPLS-DA (orthogonal partial least squares discriminant analysis) model statistics for metabolome from four tissues (comparison between genotypes of male mice). (**b**) OPLS-DA of liver metabolomic data for male and female *Aldh1l1*^+/+^ and *Aldh1l1*^−/−^ genotypes. (**c**) Comparison of selected metabolites relevant to folate and glycine metabolism.

We evaluated the effect of the *Aldh1l1* gene loss on the overall metabolic profile in the liver, brain, lung and pancreas of male mice (Supplementary Data Files 1 and 2). We have performed targeted metabolomics analysis to compare changes in common metabolites between *Aldh1l1*^−/−^ and *Aldh1l1*^+/+^ male mice (the summary of the analysis is shown in Supplementary Table S1). We have also performed metabolomic analysis in the livers of female mice. Our analysis included 334 named metabolites detected in liver, 237 in brain, 357 in lung and 318 in pancreatic tissue. OPLS-DA models with good statistics were generated for all tissues (Fig. 3a,b and Supplementary Fig. S4). However, top 25 metabolites discriminating *Aldh1l1*^−/−^ and *Aldh1l1*^+/+^ male mice were different between liver and other tissues (Supplementary Table S2; three metabolites out of 25 were common between liver and lung and one metabolite between liver and brain or pancreas). We focused further analysis on the liver metabolome (OPLS-DA for liver shows good segregation between *Aldh1l1*^−/−^ and *Aldh1l1*^+/+^ genotypes for both male and female mice, Fig. 3b). This analysis indicated that KO mice experience functional folate deficiency, the conclusion based on the accumulation of formiminoglutamate (FIGLU), which was increased about 11-fold in livers of KO mice (Fig. 3c). FIGLU is the intermediate in the folate-dependent histidine degradation pathway^27^ and a marker of folate deficiency^28^. Dihydrofolate was also strongly depleted (about 3.5-fold) in these animals (Fig. 3c), which is in agreement with folate deficiency and with the overall decrease in reduced folate pools identified by the ternary complex assay (Fig. 2). These data also indicated alterations in glycine metabolism (the drop of glycine as well as glycine conjugates with fatty acids, Fig. 3c). Of note, similar changes in these metabolites were observed in livers of both male and female mice (Fig. 3c).

**Figure 4:**
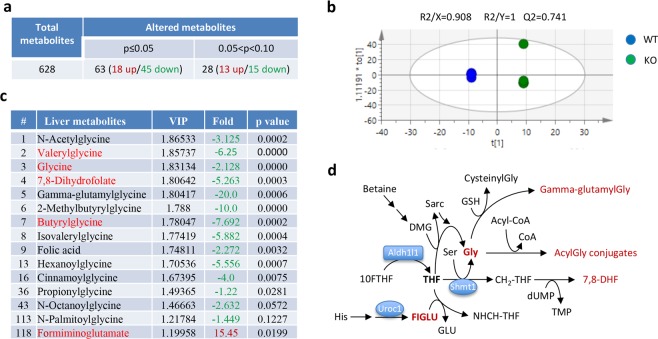
Second metabolomic analysis of *Aldh1l1*^+/+^ and *Aldh1l1*^−/−^ male mice. (**a**) Summary of identified metabolites. (**b**) OPLS-DA of genotypes. (**c**) Metabolites differentiating *Aldh1l1*^+/+^ and *Aldh1l1*^−/−^ genotypes. (**d**) Pathways of folate and glycine metabolism. Shmt1, serine hydroxymethyltransferase 1; Uroc1, urocanase 1 (urocanate hydratase 1).

To confirm our findings, the second metabolomics analysis of liver tissues from male mice was performed using higher sample numbers (5 samples per group, Supplementary Data File 3). This analysis has extended the number of named metabolites to 628 with the difference for 91 metabolites being statistically significant between the two genotypes (31 compounds being decreased and 60 being elevated in *Aldh1l1*^−/−^ compared to the *Aldh1l1*^+/+^ mice, Fig. 4a). PCA has shown good segregation between the two genotypes (Supplementary Fig. S5) and the OPLS-DA model had good statistics (Fig. 4b). This analysis confirmed findings from the previous experiment that KO mice show metabolic signs of functional folate deficiency. Specifically, FIGLU was increased more than 15-fold in KO mice (Fig. 4c). Furthermore, tissue folic acid and dihydrofolate were strongly depleted (more than 2-fold and 5-fold, respectively) in these animals, which would be in agreement with folate deficiency (Fig. 4c).

Similar to the first metabolomic experiment, a 2-fold statistically significant decrease in glycine was detected in *Aldh1l1*^−/−^ compared to the *Aldh1l1*^+/+^ mice (Fig. 4c). Strong drop in numerous glycine conjugates was also observed in the KO genotype (Fig. 4c and Supplementary Table S2), which was interpreted as the overall shortage of glycine in *Aldh1l1*^−/−^ mice. In fact, the top nine compounds differentiating *Aldh1l1*^−/−^ mice from *Aldh1l1*^+/+^ mice were glycine, glycine conjugates and two folate metabolites, with four of those compounds also identified in our first metabolomic analysis (Fig. 4c). Additional glycine conjugates were also decreased in livers of knockout mice with VIP values indicating their strong contribution to the metabolic differentiation of the two genotypes (Fig. 4c). The depletion of glycine was likely caused by the decrease of the THF-dependent synthesis of glycine from serine (schematically depicted in Fig. 4d), with the underlying basis for this phenomenon being the decrease of the THF pool observed in *Aldh1l1*^−/−^ mice (Fig. 2). In agreement with this mechanism, we also observed statistically significant elevation of serine in livers of the KO mice (fold-change was 1.38, p = 0.872, VIP was 1.14). Our data indicate the direct effect of the ALDH1L1 loss on glycine metabolism in liver.

### Effects of the ALDH1L1 loss on the gene expression profile

**Figure 5:**
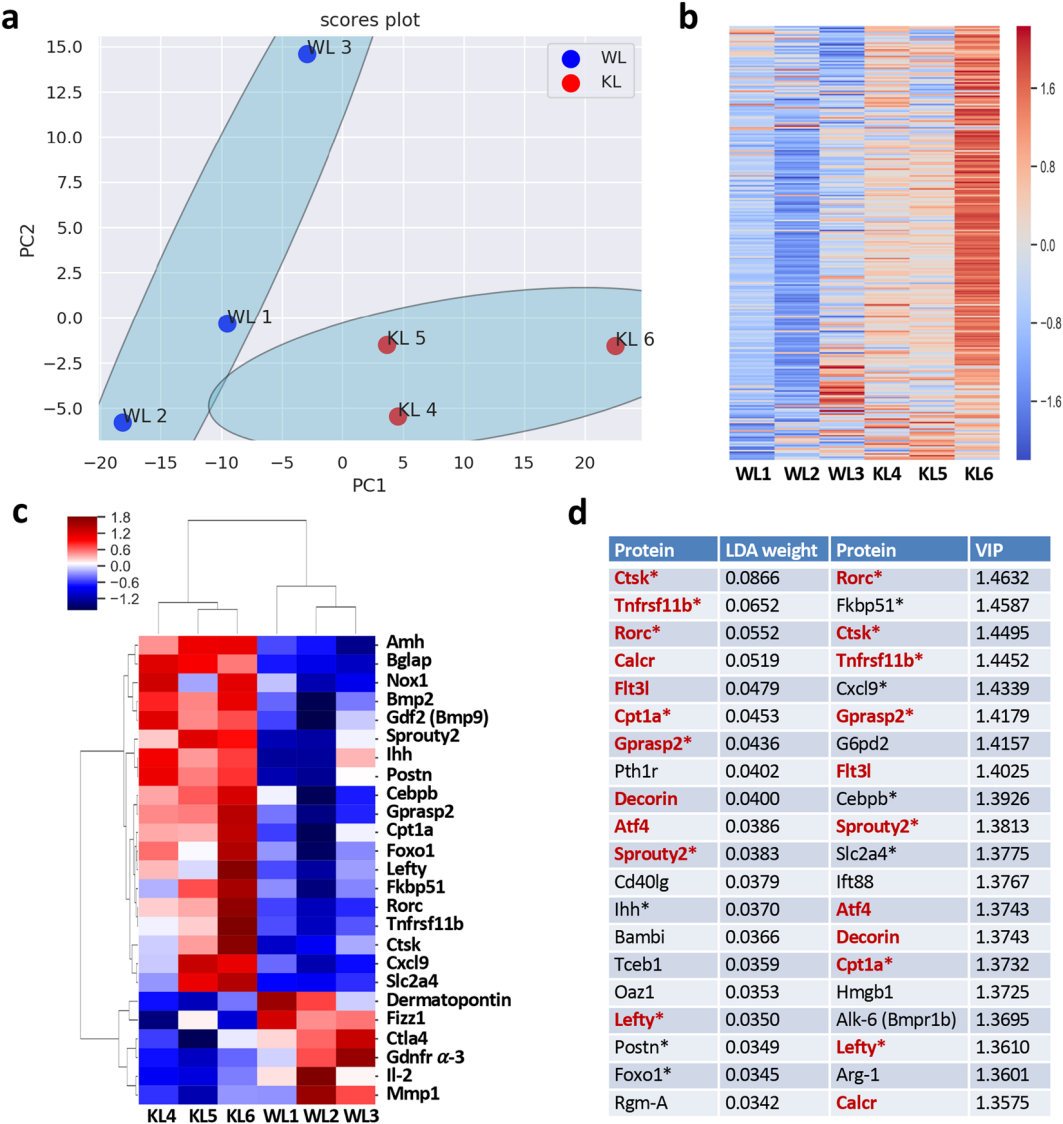
Bioinformatics analysis of NanoString data for livers of *Aldh1l1*^+/+^ and *Aldh1l1*^−/−^ male mice. Before LDA and PLS-DA analysis, data for each transcript was standardized by mean-centering and then division by the standard deviation of the message. PCA and LDA was carried out using the *sklearn* package in Python. PLS-DA analysis was carried out using the package *mixOmics* in R. (**a**) PCA for *Aldh1l1*^+/+^ (WL1-3) and *Aldh1l1*^−/−^ (KL4-6) mice. PC1 and PC2 explain 61.7% and 17.9% of the total variance of the data, respectively. (**b**) Heat map representation of the NanoString data. (**c**) A univariate analysis of targets differentially expressed in *Aldh1l1*^+/+^ and *Aldh1l1*^−/−^ mice based on the fold-change >1.5 and p-value < 0.05 cut-off. (**d**) Top 20 discriminating proteins according to LDA weights and PLS-DA VIP values. Proteins in *red* are discriminative by both LDA and PLS-DA. *Stars* indicate proteins from the list of top 25 targets (from panel **c**) differentially expressed in two genotypes. WL1-3, samples from 3 *Aldh1l1*^+/+^ mice; KL4-6, samples from 3 *Aldh1l1*^−/−^ mice.

Since ALDH1L1 protein has been established as a regulator of proliferation^12,19^, its loss is expected to evoke a cellular response with regard to altered gene expression. The diminished antioxidant pool in livers of *Aldh1l1*^−/−^ mice (Supplementary Data File 3) raised the question of whether the loss of ALDH1L1 specifically affected the expression of genes associated with inflammation. In fact, *ALDH1L1* is one of the most strongly downregulated genes in hepatocellular carcinoma in humans^20^, a typical inflammation-related cancer^29^. To investigate the effect of *Aldh1l1* knockout on gene expression profile, we performed customized NanoString analysis of mRNAs in 3-months-old male mice (Supplementary Data File 4). This panel includes a total of 242 genes relevant to inflammation and the immune response. PCA has shown that the two genotypes are well separated (Fig. 5a). The heat map (Fig. 5b) illustrates the strong difference between wild type and KO mice with regard to the expression level of the proteins included in the panel. Overall, expression levels of numerous tested targets were higher in the KO mice (Fig. 5b). A univariate analysis of targets based on the fold-change >1.5 and p-value < 0.05 cut-offs allowed the identification of a group of 25 genes most differentially expressed between the two groups (Fig. 5c). To further identify transcripts that contribute most to the discrimination between the wild type and knock out mice, we have performed a multivariate analysis applying two different supervised machine learning methods to the standardized data. Unlike the unsupervised PCA, these two methods, Linear Discriminant Analysis (LDA) and Partial Least Squares-Discriminant Analysis (PLS-DA), take advantage of the known sample type information and can quantify the relative contribution of each protein to the separation of the wild type and knockout mice^30,31^. LDA uses the coefficients/weights and PLS-DA uses the VIP values, respectively, for this purpose. Figure 5d shows the top 20 discriminating proteins based on LDA and PLS-DA analysis. Cross-reference of the genes identified by different approaches has defined a subset of genes differentially expressed at the mRNA level in *Aldh1l1*^−/−^ compared to *Aldh1l1*^+/+^ mice (Fig. 5d). A significant portion of these genes overlaps with the top 25 targets identified by the univariate analysis. Among these genes were growth factors, transcription factors, and inflammatory and immune response markers. Overall, these data suggest that ALDH1L1 protein expression is important for the control of inflammation in the liver.

## Discussion

ALDH1L1 is an enzyme of folate metabolism with a putative regulatory function. The enzyme is present at high levels in the liver, the main organ of folate metabolism, where it constitutes about 1% of the total liver cytosolic protein^32^. High expression levels of ALDH1L1 protein were also detected in kidney and pancreas, suggesting a function for the enzyme in these organs^19,23^. Several studies have provided evidence that ALDH1L1 could be important in neural system function or development as well. Thus, an early report has demonstrated that the expression of the protein is regulated in the cerebellum of mice during development^33^. Further study has confirmed the specific midline expression of ALDH1L1 and provided evidence that this enzyme has restricted expression in the developing neural tube^34^. In fact, this study demonstrated that ALDH1L1 upregulation during central nervous system development correlates with reduced proliferation and most midline ALDH1L1-positive cells are quiescent^34^. Such expression pattern correlates with the antiproliferative effect of the enzyme observed in a cell culture system^19^. Curiously, ALDH1L1 was identified as a pan-astrocyte marker^35^. ALDH1L1 is also expressed in neurons and oligodendrocytes, and its expression is decreased in the spinal cord during postnatal maturation but is up-regulated in reactive astrocytes after neural injury or chronic neurodegeneration^36^. Therefore, the loss of ALDH1L1 could be expected to have an effect on mouse phenotype.

A report of mice lacking ALDH1L1 as the result of irradiation with fission-spectrum neutrons (NEUT2 mice) indicated that the *Aldh1l1* gene is not essential for viability in the mouse^37^. *Aldh1l1*^−/−^ mice generated in our study and NEUT2 mice have shown similar alterations in hepatic reduced folate pools with a strong accumulation of 10-formyl-THF and a significant drop in THF levels, changes consistent with the loss of the 10-formyl-THF dehydrogenase activity. In addition, the total hepatic folate was also reduced upon ALDH1L1 loss, which could be a result of decreased protection of folate coenzymes from degradation^15^. In the cell, cytosolic 10-formyl-THF can be also metabolized through two other pathways^1^: (i) two reactions of the *de novo* purine biosynthesis; and (ii) the conversion to 5,10-methylene-THF by a trifunctional enzyme MTHFD1. The latter reaction though is reversible and at normal conditions it proceeds towards 10-formyl-THF generation. Of note, MTHFD1 also generates 10-formyl-THF from THF and formate in the ATP-dependent synthetase reaction (Fig. 1e)^1^. However, our study indicates that these pathways cannot compensate for the loss of ALDH1L1 with regard to 10-formyl-THF utilization. Of note, levels of corresponding enzymes were not changed upon the ALDH1L1 loss (Fig. 1c).

In contrast to mice generated in the present study, the chromosomal deletion in NEUT2 mice has also caused the loss of additional 27 genes, including urocanase 1, the gene involved in the histidine degradation pathway^34^. This pathway donates one-carbon groups to the folate pool and thus requires THF^27^. It has been suggested that, by decreasing the overall use of THF, the deletion of the urocanase 1 gene partially compensates for the decrease of the THF pool caused by the ALDH1L1 loss, that prevents the genotype manifestation^34,38^. This might explain the lack of a severe effect of the *Aldh1l1* loss in these mice. In our study, however, *Aldh1l1*^−/−^ mice also did not show noticeable phenotypic changes compared to *Aldh1l1*^+/+^ mice. Yet, metabolomic analysis did show the accumulation of FIGLU, an intermediate of the folate-dependent histidine degradation, in livers of *Aldh1l1*^−/−^ mice. This finding suggested the impairment of this pathway in *Aldh1l1* knockout mice, that is in agreement with the low THF levels (THF is required to metabolize FIGLU, Fig. 4d). Of note, elevated FIGLU in urine is used as a marker for folate deficiency^28^. Thus, the *Aldh1l1* loss is associated with functional folate deficiency in mouse liver even though the intake of dietary folate was not limited. The blood folate levels were also not changed compared to wild-type mice. Our study indicates that in the absence of ALDH1L1 enzyme, 10-formyl-THF cannot be metabolized and accumulates creating a folate trap, the situation similar to the methyl-folate trap^39^. Consistently, the drop in THF levels could be expected to cause decreased generation of glycine from serine as well as impaired histidine degradation, two pathways strictly dependent on THF.

In our study, the loss of ALDH1L1 also had a strong effect on glycine levels as well as levels of several glycine conjugates, which were dramatically reduced in knockout animals (Figs. 3c and 4c). We interpret the decrease of the glycine conjugates as the consequence of the overall shortage of glycine. Humans obtain only about 20% of glycine from their diet while about 80% is synthesized in the body^40^. In higher animals, four biosynthetic pathways can produce glycine, with two of these pathways being folate-dependent^41,42^. The pathway from glucose to glycine includes, as the final step, the THF-dependent conversion of serine to glycine. The pathway from betaine includes two THF-dependent reactions (Fig. 4d), the conversion of (i) dimethylglycine to sarcosine and (ii) sarcosine to glycine. While all three folate-dependent reactions require THF as the acceptor of one-carbon groups, the effect of ALDH1L1 on the betaine pathway is less apparent since both of its folate-dependent reactions reside in the mitochondrion^1^. Glycine, a common non-essential amino acid, in addition to its role in protein biosynthesis is the participant of numerous metabolic pathways including purine, creatine, heme and glutathione biosynthesis^41^. Thus, the depletion of glycine could be the cause of several secondary effects on metabolism such as the decrease in glutathione, lower availability of ascorbate, impaired mitochondrial lipid metabolism, oxidative stress, and less efficient remediation of toxic products^43–48^. Glycine is also a donor of one-carbon groups to the mitochondrial folate metabolism, through the glycine cleavage system^49,50^. Since the ALDH1L1 loss decreases both glycine and THF pools, the impairment of mitochondrial folate metabolism, due to decreased glycine cleavage, could be expected. This, however, needs to be tested experimentally.

The accumulation of 10-formyl-THF in *Aldh1l1*^−/−^ mice did not lead to the increase of levels of purine nucleotides. However, the effect of ALDH1L1 on purine biosynthesis would be crucial for rapidly proliferating cells but not quiescent cells. This is in agreement with the phenomenon that the ALDH1L1 expression is down-regulated in S-phase of the cell cycle but not in quiescent cells^14^. In the liver, overwhelming majority of cells are in non-proliferating state^51^, which would explain the lack of the effect of the *Aldh1l1* KO on purine pools, and this is also in agreement with the 10-formyl-THF accumulation (otherwise 10-formyl-THF would be taken up by the purine biosynthesis). Taking into account the decrease in glycine, a substrate for purine biosynthesis, in the liver of *Aldh1l1*^−/−^ mice, the effect of ALDH1L1 on the *de novo* purine pathway is more complex and to address this question with more certainty tracer experiments should be pursued. Curiously, the NanoString array has shown a significant elevation of the ATF4 transcription factor in *Aldh1l1*^*-/-*^ mice. ATF4 is involved in the regulation of serine and glycine biosynthesis and one-carbon metabolism^52–56^, as well as biosynthesis of purines through the control of the mitochondrial folate metabolism^57^. This raises the question of whether the ATF4 up-regulation in *Aldh1l1* KO mice is a compensatory mechanism in response to glycine decrease or in response to disbalance of folate pools.

Loss of glycine conjugates in *Aldh1l1*^−/−^ mice can have a diverse downstream effect on the cell. Conjugation of glycine with carboxylic acids, for example, is an important detoxification process^48^. Glycine conjugation with mitochondrial acyl-CoAs, catalyzed by glycine N-acyltransferase, is also an essential metabolic pathway responsible for maintaining adequate levels of free coenzyme A^58^. Acylglycine conjugates by themselves can also have important functions in the cell. Thus, N-acyl glycines are structurally related to endocannabinoids and at least one of such conjugates, N-arachidonoyl glycine, is a potent inhibitor of the enzyme primarily responsible for the degradation of the endocannabinoid anandamide^59^. Furthermore, several of glycine conjugates have signaling functions in the cell^59^. Of note, glycine itself is a neurotransmitter and its decrease can have a far-reaching effects beyond metabolic alterations^60^.

Glycine is an effective cytoprotective agent. In experimental models, glycine has a protective effect against a variety of diseases including ischemia/reperfusion injury, shock, transplantation, alcoholic hepatitis, hepatic fibrosis, arthritis, and tumor and drug toxicity (reviewed in^47^). Such protective function could be a direct effect on target cells or could be mediated by inhibition of inflammatory cell activation. In fact, there is also a growing body of evidence that glycine functions as an anti-inflammatory and immunomodulatory agent, and it has been used to modulate acute systemic inflammatory responses after powerful external stimuli such as multiple trauma or sepsis^42^. In this regard, *Aldh1l1*^−/−^ mice demonstrated noticeable changes in levels of proteins involved in immune response and inflammation, a likely response to the shift in glycine metabolism. Of note, folate supplementation is associated with reduced inflammation at certain physiological conditions while folate depletion led to high expression of pro-inflammatory mediators in cultured macrophages (reviewed in^61^). The link between folate and inflammatory response is likely to play a role in NAFLD which also implicates ALDH1L1 as a part of this mechanism. Indeed, our data indicate a metabolically compromised liver function in *Aldh1l1*^−/−^ mice though it does not manifest as an apparent phenotype.

Interestingly, it has been reported that the knockout of another folate enzyme, cytosolic serine hydroxymethyltransferase (SHMT1), also did not cause an obvious phenotype^62^. In this regard, ALDH1L1 and SHMT1 catalyze two consecutive steps in the glycine pathway (Fig. 4d). In contrast to *Aldh1l1*^−/−^ mice, glycine levels were not changed in *Shmt1*^−/−^ mice^62^. One explanation of this phenomenon could be the alternative splicing of the *Shmt2* gene, which encodes mitochondrial serine hydroxymethyltransferase. The omission, during *Shmt2* gene splicing, of the exon, coding for the mitochondrial leader sequence, produces cytoplasmic isozyme of SHMT^63^. This mechanism may also account for the viability of *Shmt1*^−/−^ mice^63^. Such mechanism could be envisioned for *Aldh1l1*^−/−^ mice as well since the gene encoding mitochondrial 10-formyltetrahydrofolate dehydrogenase, *Aldh1l2*, is present in genomes of higher animals including mice and humans. The alternative splicing of the *Aldh1l2* gene, which would produce a cytosolic enzyme, is not known and our data indicate that in *Aldh1l1*^−/−^ mice there is no cytosolic 10-formyltetrahydrofolate dehydrogenase. Observed changes in THF and 10-formyl-THF pools in the liver are in agreement with this conclusion.

In summary, our study of *Aldh1l1* KO mice has shown that cytosolic 10-formyl-THF dehydrogenase is a key enzyme to supply THF for glycine biosynthesis in the liver and thus is an important component of the glycine metabolic network. These findings provide a mechanistic basis for the association between *ALDH1L1* gene and serine to glycine ratio in serum^64^. An open question remains whether dietary folate deficiency can exacerbate the metabolic effects of *Aldh1l1*^−/−^ thus causing pathologies as it has been demonstrated for *Shmt1* KO mice^65^.

## Methods

### Generation of *Aldh1l1* knockout mice

All animal experiments were conducted in strict accordance with the National Institutes of Health’s “Guide for Care and Use of Laboratory Animals” and were approved by the Institutional Animal Care and Use Committee at the Medical University of South Carolina (MUSC), Charleston, South Carolina and by the Institutional Animal Care and Use Committee at the David H. Murdock Research Institute (DHMRI), Kannapolis, North Carolina. Mice were housed in microisolator cages on a 12-h light/dark cycle and allowed access to water and chow ad libitum.

ES cells (clone EPD0132_6_G09, vial #288863; *Aldh1l1* targeted, KO first) were obtained from the NCRR-NIH supported KOMP Repository (www.komp.org). This clone was from the C57BL/6N background with the agouti gene engineered into the agouti locus. Mice were generated at the MUSC Transgenic and Genome Editing Core Facility. ES cells (passage 4) have been expanded and injected into freshly isolated C57BL/6-TyrC blastocysts (10 ES cells per blastocyte), which were further injected into CD-1 pseudo-pregnant mice (12 blastocysts per mouse). Twelve chimeric pups were born from three injected females and at weaning we obtained five male chimeric mice, four having 85–99% and one 40% of the black coat color. All male chimera were bred to C57BL/6-TyrC females and black pups (both male and female) were tested for germline transmission of the targeted gene. All of the chimeras showed germline transmission. Heterozygotes for the targeted allele were bred back to the C57BL/6N mice. Heterozygous (*Aldh1l1*^+/−^) males and females were bred to obtain knockout and wild type littermates for the study.

### Genotyping

Genotyping was carried out by polymerase chain reaction (PCR) of tail lysates obtained using direct PCR (Tail) lysis reagent (cat #101-T) and Proteinase K (Specific activity >600 U/ml, Thermo Scientific, cat #EO0491). Primers for genotyping are shown in Supplementary Table S3. Amplification generates 199 bp amplicon for the WT allele and 685 bp amplicon for the mutant allele. Heterozygous males and females were intercrossed to obtain knockout and wildtype littermates.

### Western blot assays

Total protein was prepared from flash frozen tissues. Approximately 300 mg tissue was minced and homogenized in 1 ml of RIPA or IP-lysis buffer (Thermo Scientific, Pittsburg, PA) with protease and phosphatase inhibitors (1:100, Sigma-Aldrich, St. Louis, MO). Proteins were resolved by SDS-PAGE in 4–15% gradient gels (Novex, Invitrogen, Carlsbad, CA) and transferred to PVDF membranes (Millipore, Bedford, MA) in transfer buffer containing 10% methanol. Membranes were probed with primary antibodies in Tris-buffered saline with Tween- 20 containing 5% nonfat milk or BSA. A detailed description of the primary antibodies is provided in Supplementary Table S4. Horseradish peroxidase–conjugated secondary antibodies (GE Healthcare) were used at 1:5000 dilution and signal assessed with Super Signal West Pico chemiluminescence substrate (ThermoFisher Scientific, Waltham, MA, USA).

### Preparation of mitochondrial and cytosolic fractions

Mitochondria were isolated by differential centrifugation using the Mitochondria Isolation kit (Abcam, Cambridge, UK) according to the manufacture’s protocol and were solubilized using IP-lysis buffer. Following the isolation of mitochondria, the supernatant representing the cytosol was spun at 22,000 × g for 30 min and the resultant supernatant was concentrated five-fold in a centrifugal concentrator with a molecular weight cut-off of 10,000 (Merck-Millipore, Burlington, MA).

### Assays of reduced folate pools

Samples were prepared essentially as we described^66^. Fifty mg of tissues were homogenized on ice in 1 ml of 50 mM Tris-HCl buffer, pH 7.4 containing 50 mM sodium ascorbate using a Dounce homogenizer and lysed by heating for 3 min in a boiling water bath. In the case of whole blood, 50 μl of sample were mixed with 450 μl of the same buffer and treated as above. Lysates were chilled on ice and centrifuged for 5 min at 17,000 × g at 4 °C. Folate pools were measured in tissue lysates by the ternary complex assay method as we described previously^25,26^. Folate levels were calculated per milligram of protein measured by Bradford assay or per milliliter of whole blood. Statistical analysis was carried out using GraphPad Prism VIII software. Statistical significance was calculated using Student’s t-test.

### Metabolomic analysis

Metabolomics was performed using commercial services from Metabolon (Durham, NC). Individual samples (100–200 mg of flash frozen tissue) were subjected to methanol extraction then split into aliquots for analysis by ultrahigh performance liquid chromatography/mass spectrometry (UHPLC/MS). The global biochemical profiling analysis comprised of four unique arms consisting of reverse phase chromatography positive ionization methods optimized for hydrophilic compounds (LC/MS Pos Polar) and hydrophobic compounds (LC/MS Pos Lipid), reverse phase chromatography with negative ionization conditions (LC/MS Neg), as well as a HILIC chromatography method coupled to negative (LC/MS Polar)^67^. All of the methods alternated between full scan MS and data dependent MSn scans. The scan range varied slightly between methods but generally covered 70–1000 m/z. Metabolites were identified by automated comparison of the ion features in the experimental samples to a reference library of chemical standard entries that included retention time, molecular weight (m/z), preferred adducts, and in-source fragments as well as associated MS spectra and curated by visual inspection for quality control using software developed at Metabolon. Identification of known chemical entities was based on comparison to metabolomic library entries of purified standards^68^.

### Statistical analysis

Analysis was carried out essentially as described^69–72^. Two types of statistical analyses were performed: (1) significance tests and (2) classification analysis. Standard statistical analyses were performed in Array Studio software package on log‐transformed data. For analyses not standard in Array Studio, the R program (http://cran.r-project.org/) was used. Following log transformation and imputation of missing values, if any, with the minimum observed value for each compound, Welch’s two-sample t-test was used as significance test to identify biochemicals that differed significantly (p < 0.05) between experimental groups. An estimate of the false discovery rate (q‐value) was calculated to take into account the multiple comparisons that normally occur in metabolomic‐based studies. Classification analyses used included principal components analysis (PCA), hierarchical clustering, and OPLS-DA. For the scaled intensity graphics, each biochemical in the original scale (raw area count) was rescaled to set the median across all samples equal to 1.

### NanoString

RNA was extracted using a mRNeasy micro kit (Qiagen, Valencia, CA, Catalog #217084), and the gene expression analysis was performed by the MUSC deep sequencing and microarray core facility using a custom array and nCounter multiplex analysis (NanoString nCounter Technologies, Inc., Seattle, WA). Raw intensity values (counts) obtained from the analysis were normalized using counts for six housekeeping genes included in the array.

### Histological examination

Three-month-old mice were euthanized using CO_2_, organs were harvested, fixed using 4% paraformaldehyde in sodium phosphate buffer pH 7.4, and embedded in paraffin. Five-micrometer sections were stained with H&E. Examined organs were liver, spleen, kidney, heart and lungs.

## Supplementary information

Supplementary Information

Supplementary File 1

Supplementary File 2

Supplementary File 3

Supplementary File 4

## Data Availability

All data generated or analyzed during this study are included in this published article or its supplementary information files.
