# Supplementary material for: Cytosolic 10-formyltetrahydrofolate dehydrogenase regulates glycine metabolism in mouse liver
Source: Sci Rep. 2019 Oct 17;9:14937. doi: 10.1038/s41598-019-51397-1 (PMC6797707; doi:10.1038/s41598-019-51397-1)
Supplement: Supplementary file 1 — Supplementary Information [file 41598_2019_51397_MOESM1_ESM.pdf]

Cytosolic 10-formyltetrahydrofolate dehydrogenase regulates glycine metabolism in mouse liver

Natalia I. Krupenko[1,2], Jaspreet Sharma[1], Peter Pediaditakis[1], Baharan Fekry[1,#], Kristi L. Helke[3], Xiuxia Du[4], Susan Sumner[1,2] and Sergey A. Krupenko[1,2]*

[1]Nutrition Research Institute, University of North Carolina, Chapel Hill, NC, USA

[2]Department of Nutrition, University of North Carolina, Chapel Hill, NC, USA

[3]Department of Comparative Medicine, Medical University of South Carolina, Charleston, SC, USA

[4]Department of Bioinformatics & Genomics, UNC Charlotte, Charlotte, NC, USA

[#]Present address: Institute of Molecular Medicine, McGovern Medical School, University of Texas Health Science Center, Houston, TX, USA

*To whom correspondence should be addressed: sergey_krupenko@unc.edu

SUPPLEMENT (Krupenko et al)

1. Supplementary figures S1-S5

2. Supplementary Tables S1-S4

Supplementary Data Files 1-4 (separate Excel files)

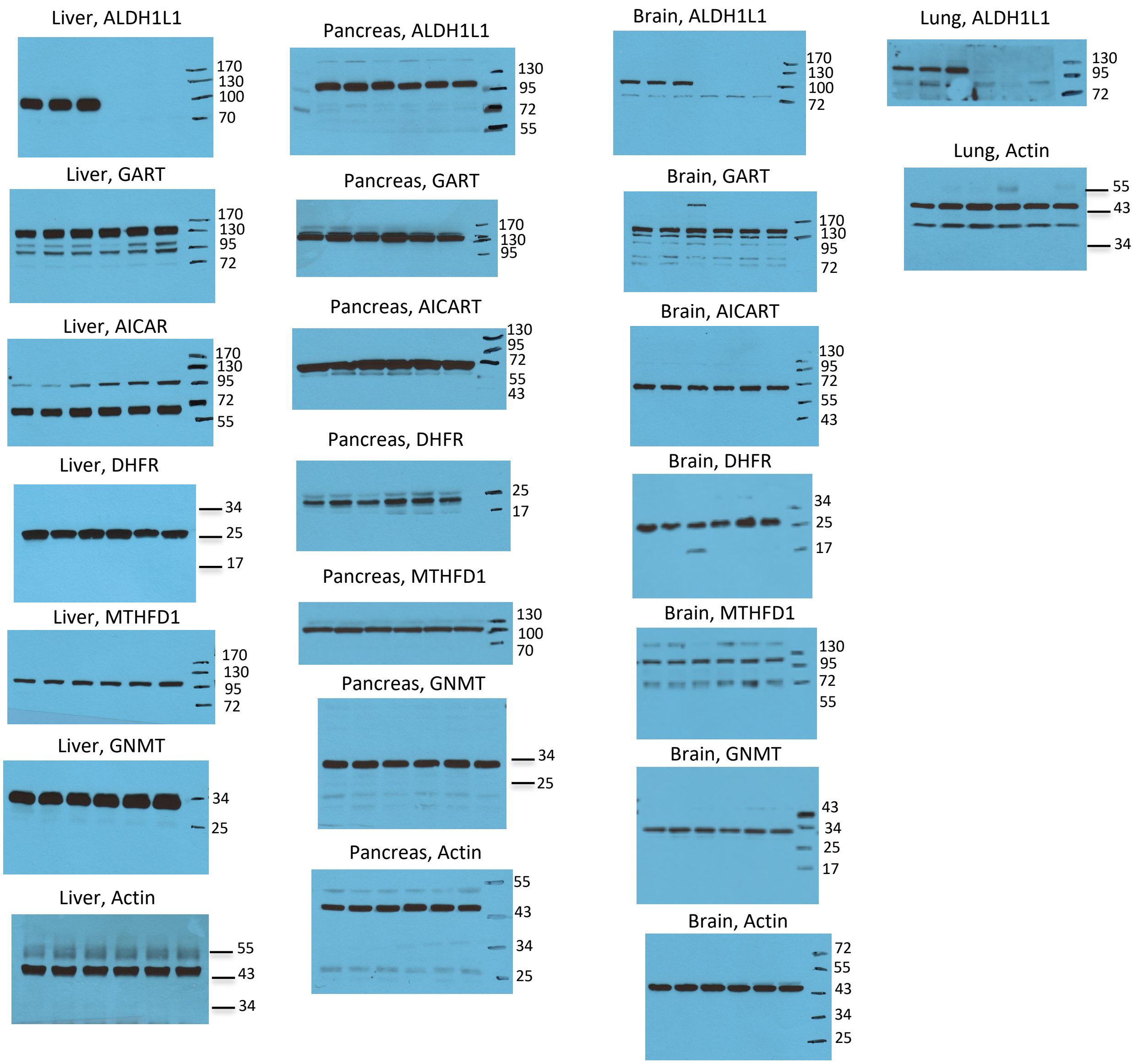

Supplementary Fig. S1. Original full-size blot images used to generate panels in Fig. 1c. Molecular mass standards were from Fisher Scientific (EZ-Run pre-stained protein ladder) or VWR (pre-stained Protein Marker IV).

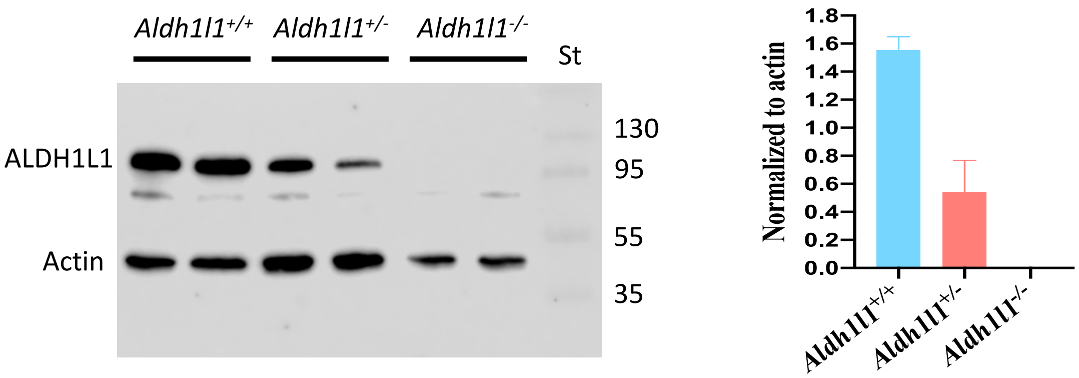

Supplementary Fig. S2. Western blot assay of liver tissues from *Aldh1l1*$^{+/+}$, *Aldh1l1*$^{+/-}$ and *Aldh1l1*$^{-/-}$ mice (two mice per genotype were analyzed). Graph shows calculation of band intensity relative to actin (Image J). Molecular mass of standards is shown. ALDH1L1 and actin were visualized using the same membrane by sequential staining with ALDH1L1-specific antibody and then with actin-specific antibody.

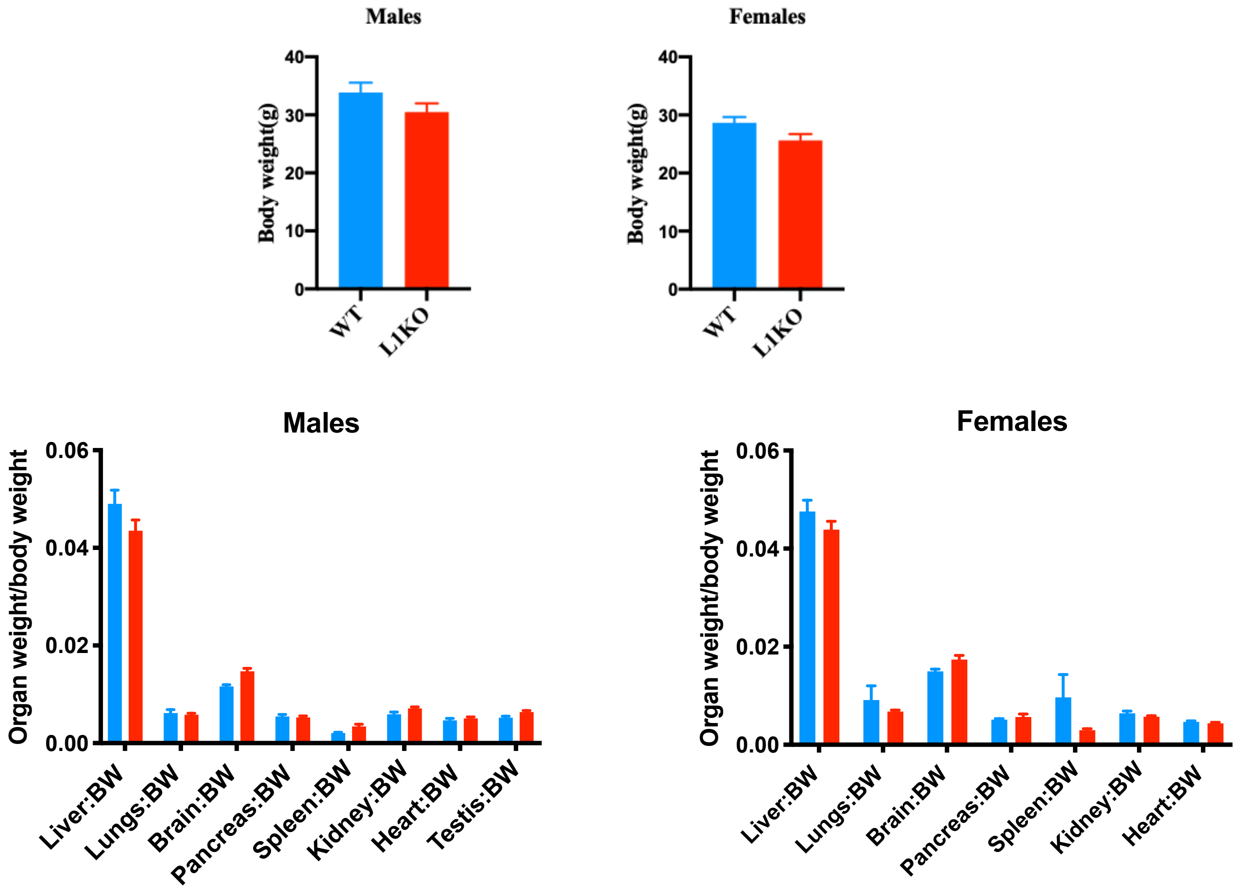

Supplementary Fig. S3. *ALDH1L1* knockout mice have body and organ weights similar to that of wild-type littermates (at 6-mo of age). *Top panels*, body weight of male and female *ALDH1L1*$^{-/-}$ (KO) and *ALDH1L1*$^{+/+}$ (WT) mice. *Bottom panels*, organ to body weight ratios for *ALDH1L1*$^{-/-}$ (*red*) and *ALDH1L1*$^{+/+}$ (*blue*) mice revealed no significant differences in organ weight. Means ± SE are shown, n=11-15 mice in each group.

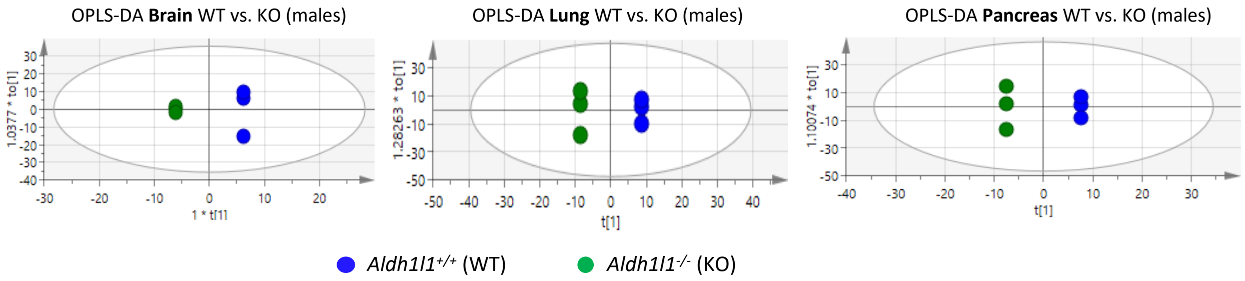

Supplementary Fig. S4. OPLS-DA for metabolites measured in tissues of *Aldh1l1*$^{+/+}$ and *Aldh1l1*$^{-/-}$ male mice.

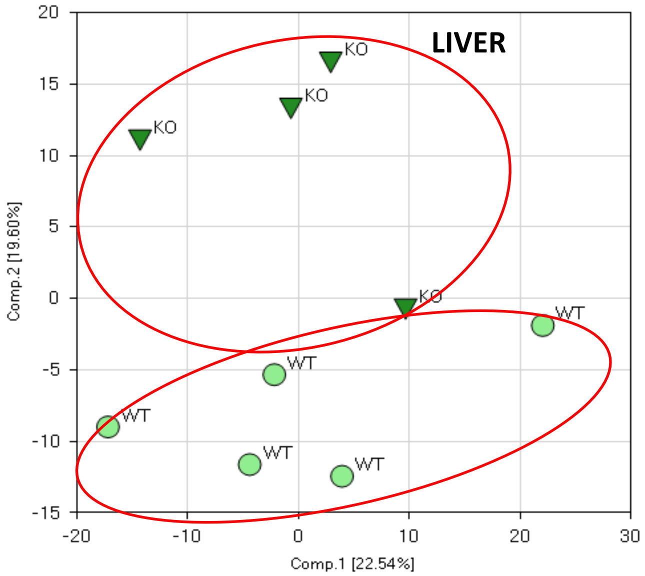

Supplementary Fig. S5. PCA for metabolites measured in liver of *Aldh1l1*$^{+/+}$ and *Aldh1l1*$^{-/-}$ male mice.

Supplementary Table S1. Statistical summary of the metabolome analysis. Analyzed datasets included total of 334 named biochemicals detected in liver tissue, 237 in brain, 357 in lung and 318 in pancreas.

<table>
<tr><th colspan="3">Statistical Summary - Liver Tissue</th></tr>
<tr><th rowspan="2">ANOVA Contrasts</th><th colspan="2">ALDH-L1 KO
WT</th></tr>
<tr><th>Female</th><th>Male</th></tr>
<tr><td>Total biochemicals
p≤0.05</td><td>42</td><td>12</td></tr>
<tr><td>Biochemicals
(↑↓)</td><td>19|23</td><td>6|6</td></tr>
<tr><td>Total biochemicals
0.05<<i>p</i><0.10</td><td>34</td><td>12</td></tr>
<tr><td>Biochemicals
(↑↓)</td><td>15|19</td><td>9|3</td></tr>
</table>

<table>
<tr><th colspan="4">Statistical Comparisons
Welch's Two-Sample t-Test</th></tr>
<tr><th rowspan="2">Significantly Altered Biochemicals</th><th colspan="3">ALDH-L1 KO
WT</th></tr>
<tr><th>Brain</th><th>Lung</th><th>Pancreas</th></tr>
<tr><td>Total biochemicals
p≤0.05</td><td>7</td><td>15</td><td>14</td></tr>
<tr><td>Biochemicals
(↑↓)</td><td>6|1</td><td>4|11</td><td>9|5</td></tr>
<tr><td>Total biochemicals
0.05<<i>p</i><0.10</td><td>6</td><td>11</td><td>11</td></tr>
<tr><td>Biochemicals
(↑↓)</td><td>1|5</td><td>1|10</td><td>7|4</td></tr>
</table>

Supplementary Table S2. Top 25 metabolites differentiating metabolome of *Aldh1l1*$^{+/+}$ and *Aldh1l1*$^{-/-}$ male mice in 4 tissues (VIP, variable importance of the projection).

#	Liver metabolites	VIP	Brain metabolites	VIP	Lung metabolites	VIP	Pancreas metabolites	VIP
1	valerylglycine	1.71466	serine	1.73273	caprylate (8:0)	1.56588	gamma-glutamylglutamate	1.64056
2	N-acetylglucosamine 6-phosphate	1.70719	pelargonate (9:0)	1.71731	indolepropionate	1.56108	nonadecanoate (19:0)	1.62528
3	dehydroascorbate	1.67441	cystathionine	1.69777	nicotinamide adenine dinucleotide (NAD+)	1.54602	coenzyme A	1.60972
4	gamma-glutamyltyrosine	1.62415	palmitoyl sphingomyelin	1.61386	flavin mononucleotide (FMN)	1.53983	erucate (22:1n9)	1.55597
5	tauroursodeoxycholate	1.61838	hydroxybutyrylcarnitine	1.56958	uridine	1.53453	17-methylstearate	1.53123
6	butyrylglycine	1.59562	S-adenosylhomocysteine (SAH)	1.56165	stearidonate (18:4n3)	1.5127	creatinine	1.5234
7	adenine	1.57763	fructose	1.52923	glutathione, oxidized (GSSG)	1.50211	isobutyrylcarnitine	1.51782
8	glycine	1.5745	alanine	1.49133	hippurate	1.47928	gamma-glutamylthreonine*	1.51719
9	5-methylthioadenosine (MTA)	1.55481	Isobar: ribulose 5-phosphate, xylulose 5-phosphate	1.48469	cytidine 5'-diphosphocholine	1.46845	Isobar: UDP-acetylglucosamine, UDP-acetylgalactosamine	1.50863
10	7,8-dihydrofolate	1.54366	glycerol	1.47494	citrate	1.4667	isoleucine	1.5067
11	tauro(alpha + beta)muricholate	1.53646	threonine	1.46697	S-methylglutathione	1.45344	1,5-anhydroglucitol (1,5-AG)	1.49211
12	flavin mononucleotide (FMN)	1.52356	glucose-6-phosphate (G6P)	1.4662	cysteine	1.44391	arachidate (20:0)	1.49052
13	1,5-anhydroglucitol (1,5-AG)	1.51278	3-(4-hydroxyphenyl)lactate	1.45583	tetradecanedioate	1.43795	xanthine	1.48026
14	UDP-galactose	1.51103	putrescine	1.455	nonadecanoate (19:0)	1.38562	N-acetylleucine	1.47657
15	hippurate	1.49468	succinylcarnitine	1.44688	N-acetylglycine	1.38555	13-methylmyristic acid	1.47096
16	ribose	1.48811	gamma-glutamylphenylalanine	1.44618	palmitoyl ethanolamide	1.38408	arginine	1.46807
17	1,3-dihydroxyacetone	1.48782	propionylcarnitine	1.44309	corticosterone	1.36309	glycerophosphorylcholine (GPC)	1.45631
18	pantethine	1.48709	1-palmitoylplasmenylethanolamine	1.43719	succinylcarnitine	1.35569	choline phosphate	1.44615
19	riboflavin (Vitamin B2)	1.47801	pentadecanoate (15:0)	1.42876	putrescine	1.35469	nicotinamide adenine dinucleotide (NAD+)	1.44519
20	taurohyodeoxycholic acid	1.47357	methionine	1.42666	margarate (17:0)	1.34248	gamma-glutamylvaline	1.42871
21	N-acetylserine	1.47196	ergothioneine	1.39	2'-deoxyinosine	1.34147	eicosenoate (20:1n9 or 11)	1.42154
22	6-beta-hydroxylithocholate	1.46154	cis-vaccenate (18:1n7)	1.36892	cysteine-glutathione disulfide	1.33756	hypotaurine	1.41005
23	2-hydroxystearate	1.456	deoxycarnitine	1.36277	hexadecanedioate	1.32964	propionylcarnitine	1.40681
24	citrate	1.45309	histidine	1.35717	guanosine 5'-diphospho-fucose	1.32958	10-nonadecenoate (19:1n9)	1.39998
25	serine	1.4463	cytidine-5'-diphosphoethanolamine	1.35023	maltose	1.32145	glutamate	1.38053

Supplementary Table S3. Primers used for genotyping.

Primers	Sequence	Size(bp)
WTf	5′- CCGAAGGACCAAACTCTTTCC-3′	199 bp
WTr	5′- TCCTTACAGCCCTTTCCAGAG-3′	
RAF5	5′- CACACCTCCCCCTGAACCTGAAA -3′	685 bp
TTR1	5′- AAGCTTCCTCTCTGTTCTACCCACAC-3′	

Supplementary Table S4. List of primary antibodies used for Western blot assays.

Target	Antibody	Source	Cat# or reference*	Dilution
AICART	Rabbit polyclonal	In-house	[1]	1:10,000
ALDH1L1	Rabbit polyclonal	In-house	[2]	1:10,000
Actin	Mouse monoclonal	Abcam	ab8226	1:10,000
DHFR	Rabbit monoclonal	Abcam	ab133546	1:1,000
GART	Rabbit polyclonal	In-house	[1]	1:10,000
MTHFD1	Rabbit polyclonal	In-house	[1]	1:10,000
GNMT	Rabbit polyclonal	In-house	[3]	1:4000

1 Oleinik, N. V., Krupenko, N. I., Reuland, S. N. and Krupenko, S. A. (2006) Leucovorin-induced resistance against FDH growth suppressor effects occurs through DHFR up-regulation. Biochem Pharmacol. **72**, 256-266

2 Krupenko, S. A. and Oleinik, N. V. (2002) 10-formyltetrahydrofolate dehydrogenase, one of the major folate enzymes, is down-regulated in tumor tissues and possesses suppressor effects on cancer cells. Cell Growth Differ. **13**, 227-236

3 DebRoy, S., Kramarenko, II, Ghose, S., Oleinik, N. V., Krupenko, S. A. and Krupenko, N. I. (2013) A novel tumor suppressor function of glycine N-methyltransferase is independent of its catalytic activity but requires nuclear localization. PLoS One. **8**, e70062
